# Model-Informed Precision Dosing of Antibiotics in Pediatric Patients: A Narrative Review

**DOI:** 10.3389/fped.2021.624639

**Published:** 2021-02-23

**Authors:** Alan Abdulla, Elma Edwina, Robert B. Flint, Karel Allegaert, Enno D. Wildschut, Birgit C. P. Koch, Matthijs de Hoog

**Affiliations:** ^1^Department of Hospital Pharmacy, Erasmus University Medical Center, Rotterdam, Netherlands; ^2^Division of Neonatology, Department of Pediatrics, Sophia Children's Hospital, Erasmus University Medical Center, Rotterdam, Netherlands; ^3^Department of Pharmaceutical and Pharmacological Sciences, KU Leuven, Leuven, Belgium; ^4^Department of Development and Regeneration, KU Leuven, Leuven, Belgium; ^5^Department of Pediatric Intensive Care, Sophia Children's Hospital, Erasmus University Medical Center, Rotterdam, Netherlands

**Keywords:** pediatric, neonates, antibiotics, model-informed precision dosing, Bayesian, therapeutic drug monitoring, population PK models

## Abstract

Optimal pharmacotherapy in pediatric patients with suspected infections requires understanding and integration of relevant data on the antibiotic, bacterial pathogen, and patient characteristics. Because of age-related physiological maturation and non-maturational covariates (e.g., disease state, inflammation, organ failure, co-morbidity, co-medication and extracorporeal systems), antibiotic pharmacokinetics is highly variable in pediatric patients and difficult to predict without using population pharmacokinetics models. The intra- and inter-individual variability can result in under- or overexposure in a significant proportion of patients. Therapeutic drug monitoring typically covers assessment of pharmacokinetics and pharmacodynamics, and concurrent dose adaptation after initial standard dosing and drug concentration analysis. Model-informed precision dosing (MIPD) captures drug, disease, and patient characteristics in modeling approaches and can be used to perform Bayesian forecasting and dose optimization. Incorporating MIPD in the electronic patient record system brings pharmacometrics to the bedside of the patient, with the aim of a consisted and optimal drug exposure. In this narrative review, we evaluated studies assessing optimization of antibiotic pharmacotherapy using MIPD in pediatric populations. Four eligible studies involving amikacin and vancomycin were identified from 418 records. Key articles, independent of year of publication, were also selected to highlight important attributes of MIPD. Although very little research has been conducted until this moment, the available data on vancomycin indicate that MIPD is superior compared to conventional dosing strategies with respect to target attainment. The utility of MIPD in pediatrics needs to be further confirmed in frequently used antibiotic classes, particularly aminoglycosides and beta-lactams.

## Introduction

Antibiotics are the most commonly prescribed drugs in children and are potentially life-saving for patients with severe bacterial infections (1–3). Based on a cross-sectional one-day point prevalence survey, more than 35% and 40% of hospitalized children in European and non-European countries, respectively, received antibiotics (4). However, antibiotic dosing in pediatric patients is challenging and often more complex than in adult patients.

Children have different and changing body composition, body size, physiology and body chemistry. Furthermore, there is developmental growth and maturation of organs which may contribute to the variability in the pharmacokinetics/pharmacodynamics (PK/PD) of drugs and treatment outcomes (5, 6). Consequently, age-related differences in absorption, distribution, metabolism and elimination of drugs have been demonstrated in children. For example, the expression and activity of drug-metabolizing iso-enzymes in the liver is yet immature at birth and the rate of maturation has high inter-individual variability (7, 8). This can result in a significant risk of toxicity with some drugs in neonates and infants (9). Additionally, neonates are more vulnerable to life-threatening infectious diseases, due to their immature immune system, diminished humoral response, reduced skin barrier, and low microbial variation in gut microbiota composition (10–12).

In certain pediatric populations with significant intra- and inter-patient variability, such as children with obesity, inflammation, organ failure, critical illness, or other significant co-morbidities and co-medication affecting drug exposure, conventional age or weight-based dosing regimens does not seem to be optimal (13–15). These populations can greatly benefit from individualized dosing. Dosing in pediatric patients, especially antibiotic and anticancer drug treatment, is challenging and can result in supratherapeutic exposure that potentiates undesirable side effects or toxicity (16–18), while subtherapeutic exposure can contribute to treatment failure (19, 20). Moreover, under-exposure of antibiotics may result in further emergence of drug resistance, although this relationship has not been studied in detail. These factors imply that antibiotic dosing in pediatric patients demands a thorough assessment.

In general, conventional dosing regimens of antibiotics are usually based on current body weight, age or nomograms and adjusted for renal function as needed. However, this approach is often not optimal and in some cases even not sufficient to achieve predetermined PK/PD target values (21–23). Besides, dosing recommendation from developed PK models can only address the patient population and characteristics of the cohort that was used for PK model development, thus the extrapolation to different patient characteristics is not possible. For some antibiotics, therapeutic drug monitoring (TDM) is used to optimize pharmacological target attainment and therefore decrease therapeutic failure and toxicity (24). Dose adjustments should be made in an early phase of treatment, since quick and accurate intervention with antibiotics is essential for patients with severe infections. However, a first TDM sample for antibiotics is generally requested if a steady state concentration is reached (meaning after four to five half-lives of the drug), which cannot be considered ‘early'. In order to predict those concentrations, population PK (popPK) modeling combined with early TDM sampling, before steady state, is a valuable dosing strategy to optimize antibiotic therapy. This approach includes interpreting drug concentrations along with patient information, such as age, body weight, kidney function, and dose history (25, 26). PopPK modeling combined with TDM typically covers an assessment of PK and concurrent dose adaptation alone. The concept of Model-informed precision dosing (MIPD) involves the use of popPK models and prospective Bayesian forecasting to reduce variability in response. A Bayesian approach delivers a population estimated value for each PK parameter including the variability components, that is, noise (residual error) and variability due to real biological differences between individuals (inter-individual variability), simultaneously.

### Workflow of Model-Informed Precision Dosing Implementation

**Figure 1:**
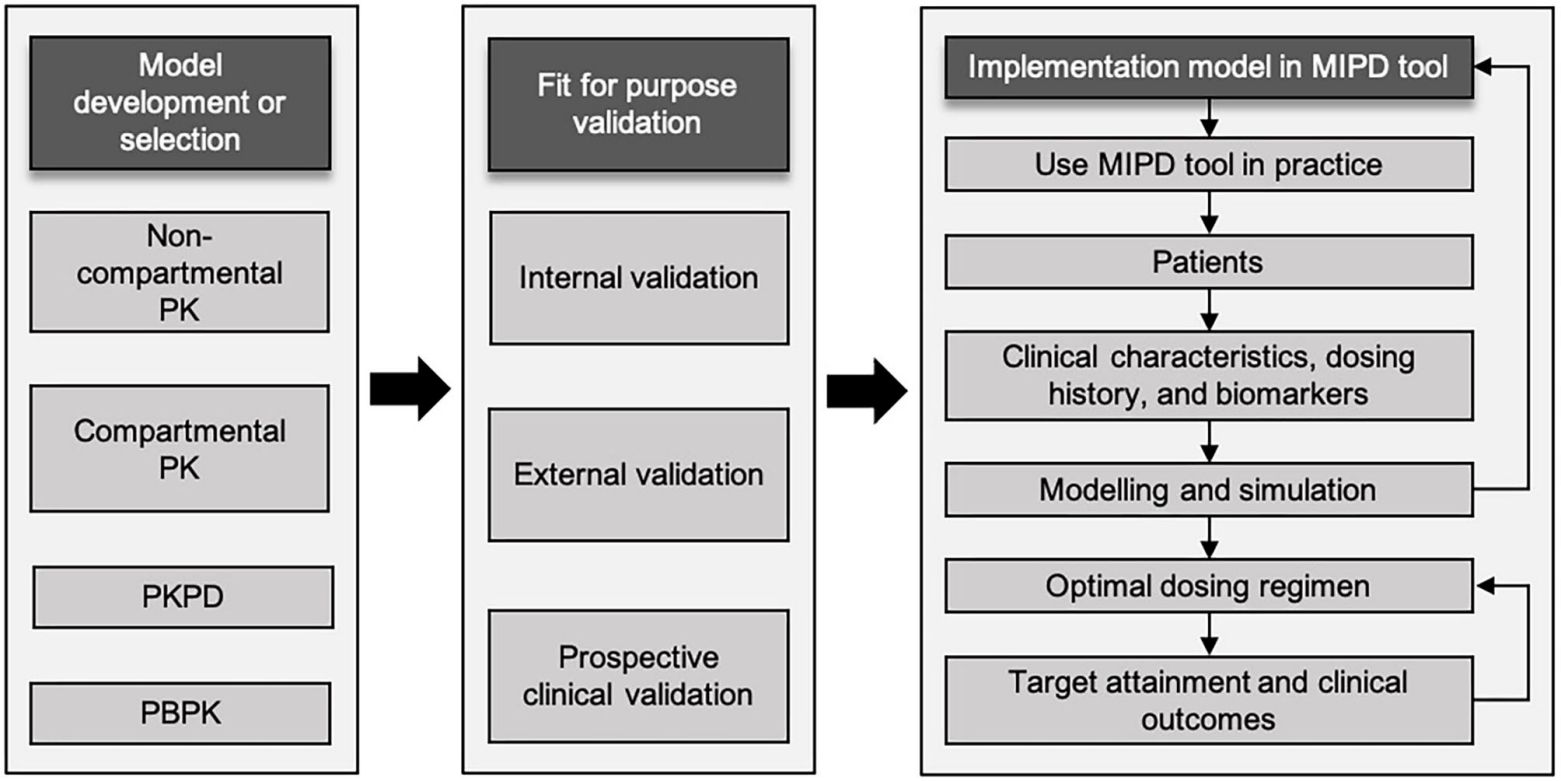
Schematic workflow of model-informed precision dosing implementation. Adapted from Darwich et al. and Keizer et al. (27, 28). MIPD, model-informed precision dosing; PBPK, physiologically based pharmacokinetics; PK, pharmacokinetics; PK/PD, pharmacokinetic/pharmacodynamics.

A workflow involving several steps has been proposed to achieve optimal dosing in children employing an MIPD approach (Figure 1) (27, 28). Firstly, an appropriate population model, including compartmental PK model, PK/PD model, or physiological-based PK (PB/PK) model, needs to be selected or developed if not available. Model development can be performed using PK/PD modeling software (e.g., NONMEM, WinNonLin, Pmetrics, or MatLab). The selected PK model should fit the population characteristics, such as age group, body composition, disease and comorbidities. Even biomarkers, for instance serum creatinine concentration to predict vancomycin concentrations, may be relevant (29). For several antibiotics, extensive modeling performances have resulted in numerous popPK models over the full pediatric age ranges (30). In contrast, some classes of antibiotics have limited models, or models that only describe a narrow age range (28). Therefore, popPK modeling is a powerful method to study PK in children due to its ability to deal with sparse and time flexible blood sampling, identification of PK variability, and dosing simulations (31). Secondly, model validation is an essential step to be conducted. Internal validation should be performed to diagnose any model misspecifications, and external validation is needed to evaluate model performance in a different cohort of patients with similar characteristics to the one used to develop the model. Although the popPK approach has been around for decades, the benefits are not always obvious to clinicians and therefore translation into clinical practice has been very limited (31). Nevertheless, there are early examples of applying MIPD in the clinical practice for carboplatin and busulfan with significant advantage over dosing strategies to achieve target exposure (32, 33). MIPD software tools are used to optimize dosing in both initial and subsequent treatment regimens combined with TDM (34). The aim is to achieve drug exposure targets in each individual patient as soon as possible, that is, to achieve drug concentration related to minimal inhibitory concentration (MIC) and at the same time avoiding toxicity and side effects. Finally, all these steps need to be followed to embed a validated popPK model in an MIPD software tool (35). To evaluate the benefits of MIPD approach in clinical practice, prospective clinical validation in the population of interest should be conducted (27).

### Model-Informed Precision Dosing Implementation in Clinical Practice

Although numerous MIPD software tools have been developed over the past decades, they have still not been widely integrated into clinical practice (34). MIPD implementation of modeling strategies can be divided into three categories: (1) real-time implementation of MIPD models aligned in healthcare; (2) mechanistic modeling and extrapolation based on prior information on patient characteristics; (3) and model-derived dose banding from covariate analysis of large population studies (27). An overview and description of available MIPD software tools is detailed in Table 1. These software tools performed well with respect to all evaluated categories (34). To bring MIPD in the clinical practice, the integration of Electronic Health Record (EHR) and Clinical Decision Support (CDS) is considered as the best approach for clinical adaptation (36). The MIPD approach has been used to optimize dosing in the adult population with significant improvements (37). Likely, MIPD is a promising option to enhance drug efficacy and safety using integration of real-time patient data and it may play an important role in the wider context of precision medicine. In this narrative review, we evaluate studies assessing the clinical utility of antibiotic pharmacotherapy using MIPD in pediatric populations.

**Table 1:** Descriptive characteristics of the model-informed precision dosing software tools.

**MIPD software tools**	**Company/Institution**	**User platform**	**Purpose of use**	**Mathematical software**	**EHR-integrated**	**Overall performance (%)** ^*^
Autokinetics	Departments of intensive care medicine of Amsterdam UMC	Desktop, web-based	Research and clinical	NONMEM^®^, R^®^	Yes	68
Bestdose	Laboratory of Applied Pharmacokinetics and Bioinformatics, Children's Hospital Los Angeles	Desktop, Web-based	Research	-	No	54
DoseMeRx	DoseMe (Tabula Rasa HealthCare Company)	Web-based, android and iOS	Research and clinical	GNU Scientific Library	Yes	78
ID-ODS	Optimum Dosing Strategies	Web-based, android and iOS	Clinical	Matlab^®^	No	74
InsightRX Nova	Insight Rx Inc.	Web-based	Research and clinical	NONMEM^®^	Yes	83
MwPharm++	Mediware a.s.	Desktop, Web-based, Android, iOS	Research and clinical	-	Yes	82
NextDose	University of Auckland	Web-based	Research and clinical	-	No	66
PrecisePK	Healthware Inc.	Desktop, Web-based	Research and clinical	-	Yes	77
TDMx	Institute of Pharmacy, University of Hamburg	Web-based	Research and clinical	NONMEM^®^	No	56
Tucuxi	School of Engineering and Management Vaud	Desktop	Clinical	NONMEM^®^	Yes	57

*Data adapted from Kantasiripitak et al. (34)*.

* *These software tools were evaluated based on eight considered criteria, including user-friendliness and utilization, user support, computational aspects, population models, quality and validation, output generation, privacy, data security, and costs*.

*HER, electronic health record; MIPD, model-informed precision dosing; NONMEM, non-linear mixed effects model*.

## Methods

A literature search was conducted in September 2020 without a restriction of the publication date. Three databases (Medline All Ovid, Embase and Web of Science Core Collection) were searched to assess literature on the clinical utility of MIPD for antibiotics among the pediatric population. Detailed research terms can be found in Supplementary Table 1. Only original research articles reporting the clinical utility of MIPD for antibiotics in pediatric patients were eligible for inclusion. Table 2 shows the inclusion and exclusion criteria.

**Table 2:** In- and exclusion criteria used to select relevant articles.

**Inclusion criteria**	**Exclusion criteria**
• The study was performed in neonates, children, or adolescents aged up to 18 years or included both children and adults • The study included the clinical use of model-based/informed precision dosing (MBPD/MIPD) for antibiotics • Outcome measures are reported, that is, target attainment	• Pre-clinical or non-human studies • Modeling and simulation-only studies • Non-English articles • Conference papers and abstracts

The references from the database were imported into a reference manager (Endnote X9^®^) and a published inclusion strategy was used (38). Titles and abstracts were screened independently by two reviewers (AA and AE). Disagreements were resolved by means of consensus. Relevant studies identified from references of our included articles. Even though review and expert opinion articles were excluded, the reference lists of these records were also checked. We extracted the following data: author, year of publication, study antibiotic, number of participants, age category, PK model reference, and outcome measurements, from each study included in the narrative review.

## Results

### Search Results and Selection of Articles

**Figure 2:**
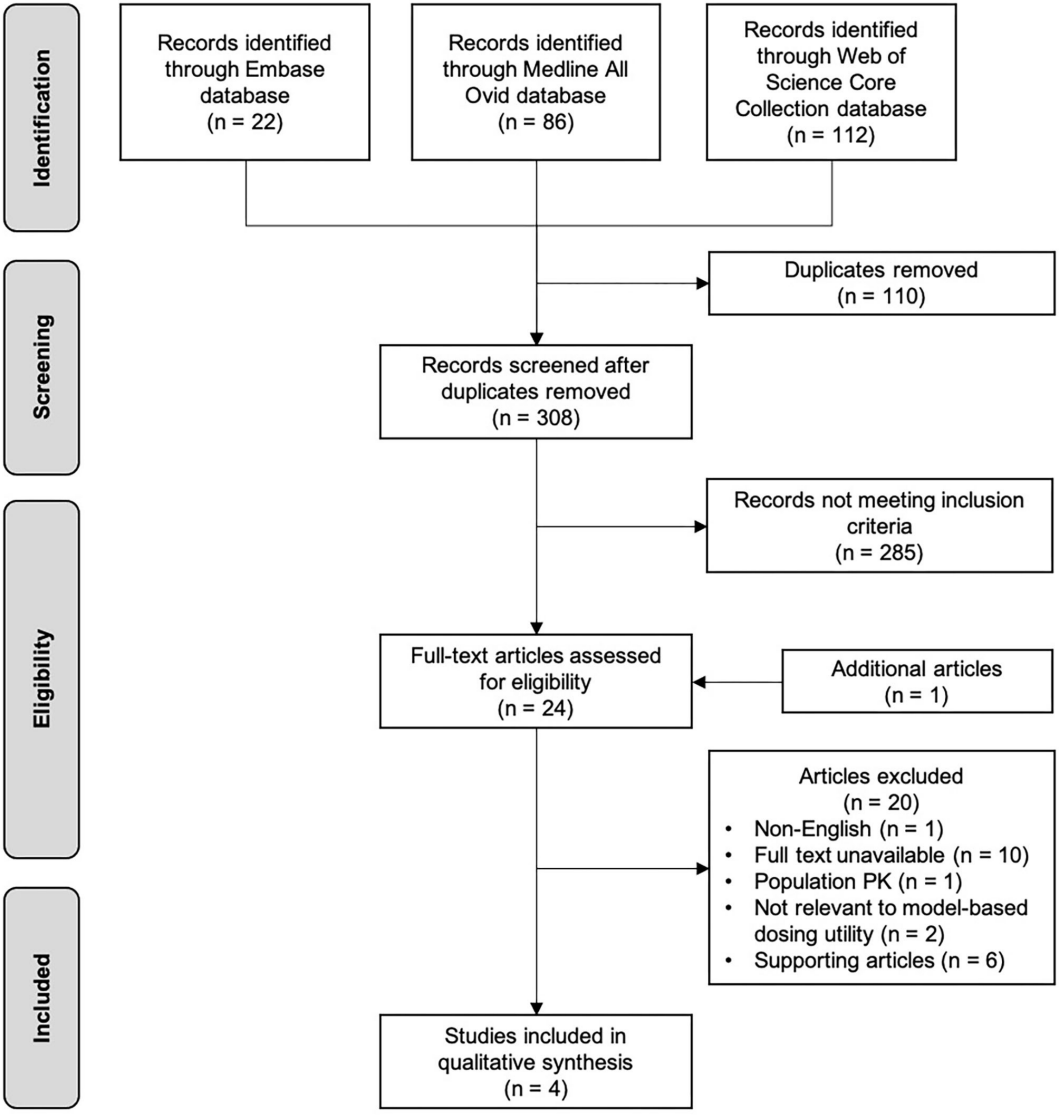
Flowchart of the selection process.

Figure 2 shows the flowchart of the selection process of this review. The initial search through databases resulted in 418 records. After removing duplicates, followed by screening titles and abstracts, 23 articles were eligible as full text assessment. One additional study was identified from reference list checking. A total of four studies were included in this narrative review (39–42), and six studies on this topic that did not meet the inclusion criteria were used to support the concept of MIPD (43–48). Table 3 shows the characteristics of the four included studies. Studies were reviewed in chronological order of the year of publication concerning evolving knowledge of MIPD.

**Table 3:** Characteristics of included studies investigating the utility of model-based precision dosing for antibiotics in children.

**First author, year**	**Study Antibiotic**	**Number of patients**	**PK model reference**	**Covariates**	**Comparison**	**MIPD category** ^*^	**Main outcome measurement**	**Other outcome measurements**	**Most superior dosing strategy**
Smits et al. (39)	Amikacin	579 neonates (1–30 days)	(46)	Current body weight, PNA	MIPD vs. population PK model	3	Early trough and peak level attainment	Toxic trough level attainment	MIPD
Leroux et al. (40)	Vancomycin	190 neonates	(49)	Current body weight, birthweight, PNA, sCr	MIPD vs. standard regimen	1	Early trough and peak level attainment	Nephrotoxicity	MIPD
Frymoyer et al. (41)	Vancomycin	492 term and preterm neonates	(50, 51)	PMA, current body weight, sCr	MIPD (Neo-Vanco) vs. Neofax, Red Book and Lexicomp	1	AUC_24h_/MIC and trough level attainment at steady state	Toxic trough level attainment	MIPD
Hughes et al. (42)	Vancomycin	144 children (1–18 years)	(52)	Age, sCr, body weight	MIPD vs. clinician judgement-guided dosing	1	Trough level attainment at steady state	AUC_24h_ attainment	MIPD

* *These categories refer MIPD implementation strategies: 1 = real-time implementation of MIPD models aligned in healthcare; 2 = mechanistic modeling and extrapolation based on prior information on patient characteristics; 3 = and model-derived dose banding from covariate analysis of large population studies*.

*AUC_24h_, the area under the concentration-time curve from 0 to 24 h; MIC, minimum inhibitory concentration; MIPD, model-informed precision dosing; PK, pharmacokinetic; PNA, post-natal age; PMA, post-menstrual age; sCr, serum creatinine*.

### Utility Studies of Model-Informed Precision Dosing in Pediatric Patients

Smits et al. (39) is the only study included in this review which investigated MIPD of amikacin, the other included studies all evaluated vancomycin. They conducted a prospective evaluation of a model-based amikacin dosing regimen in 579 neonates with postnatal age of 1–30 days. This dosing regimen was based on several covariates, including current body weight and postnatal age. Based on a popPK model (46), a simplified version of the model-based dosing regimen was applied. To evaluate the simplified age groups and dosing intervals, the percentage of desired early trough concentration (before second dose) and peak concentrations (after second dose) was considered as the main outcome measurement. The predefined targets for neonatal population were trough concentrations of 1.5–3 mg/L and peak concentrations of >24 mg/L. The simplified model-based dosing regimen resulted in better amikacin exposure, 90.5% of the observed early peak levels reached the predefined of target of >24 mg/L. Moreover, 60.2% of the trough levels were <3 mg/L. Only 6.6% of first trough concentrations were >5 mg/L. Target concentration attainment at steady state was demonstrated using Monte Carlo simulation. In almost all patients without ibuprofen co-administration the simulations resulted in adequate trough concentrations. The prospective evaluation of the model-based neonatal amikacin dosing regimen resulted in better peak and trough concentrations in almost all patients than the previously developed population PK model. Furthermore, adapted dosing was proposed for patient subgroups with suboptimal trough levels. Moreover, since about half of the neonates with a postnatal age <14 days and body weight of >2,000 g had the first trough levels already within 3–5 mg/L, the authors suggested an interval prolongation of 6 h. This result also indicated that MIPD enables further improvement of drug dosing regimens in neonates.

Similarly, Leroux et al. (40) conducted a prospective clinical trial to evaluate the clinical utility and safety of MIPD of vancomycin dosing in 190 neonates. They developed an Excel^®^ dosing calculator using a previously published population PK model (49). Covariates that were inserted to calculate tailored dosing included birth weight, current body weight, postnatal age (PNA), and serum creatinine concentration measured within 48 h after vancomycin treatment. The percentage of patients who attained the target trough concentrations (15–25 mg/L) was defined as the outcome measurement. Early TDM samples taken 6 to 24 h following the initiation of the vancomycin treatment were used. Furthermore, the authors compared the target attainment using MIPD to a standard dosing regimen based on their previous work (49). The target attainment to the trough range of 15–25 mg/L was 41% when the standard regimen was used, while the target attainment rate when using MIPD increased to 72%. In addition, the safety outcome during vancomycin therapy was nephrotoxicity, defined as either a two-fold increase or an increase by at least 0.6 mg/dL of serum creatinine concentrations from the start and any time until the end of therapy. Of the 190 neonates receiving the MIPD of vancomycin, only 2 (1.1 %) patients developed nephrotoxicity compared to 8.7% (6 of 69) of neonates receiving standard dose in the previous study (53). The elevated serum creatinine in these two patients was considered not related to vancomycin therapy. This study clearly demonstrated the potential of MIPD to increase the efficacy and safety of vancomycin dosing in neonatal routine care.

A retrospective evaluation of vancomycin MIPD was performed by Frymoyer et al. (41). They investigated the Neo-Vanco, which was designed to personalize empiric vancomycin dosing in neonates based on post menstrual age (PMA), weight and serum creatinine levels as covariates, and externally validated (50, 51). Neo-Vanco was compared to commonly used dosing guidelines, including Neofax, Red Book, and Lexicomp. The outcome measurements in their study were the probability of attaining a 24-h area under the curve/minimum inhibitory concentration ratio (AUC_24h_/MIC) of >400, and trough concentrations of 5–20 mg/L at steady state. The percentage of neonates predicted to achieve an AUC_24h_/MIC of >400 target was 94% with Neo-Vanco, 18% with Neofax, 23% with Red Book, and 55% with Lexicomp (all *P* <0.0001 vs. Neo-Vanco). Furthermore, a trough concentration of <5 mg/L was observed infrequently in neonates for whom Neo-Vanco was used, whereas a trough concentration of <5 mg/L was predicted to occur more often with the other dosing strategies (all *P* < 0.0001 vs. Neo-Vanco). Extremely high trough concentrations of >20 mg/L occurred only in 2.8% of neonates with Neo-Vanco this was similar across the dosing approaches (Neofax 1.0% (*P* = 0.030), Red Book 2.6% (*P* = 0.99), and Lexicomp 4.1% (*P* = 0.27). Overall, results indicate that target exposure levels were attained more consistently with Neo-Vanco. Additionally, this model-based dosing approach allows the incorporation of drug-concentration data and can be used to support AUC_24h_/MIC predictions and dose adjustments.

The third MIPD of vancomycin study was performed by Hughes et al. who conducted retrospective evaluation based on simulations (42). In this study, in 144 children aged 1–18 years a clinical decision support (CDS) dose-optimizing software program was compared with clinician judgement in individualizing vancomycin dosing regimens. InsightRX, a website platform and CDS tool, was used in this study. The aims were to integrate PK/PD models with Bayesian forecasting of drug concentrations, and to evaluate personalized dosing. A previously published population PK model was used for model fitting and simulations of concentration-time profiles (52). Depending on serum creatinine, age and current body weight, the model-based dosing was determined. Similar to the study of Frymoyer et al. (41), the primary outcome measurement was the number of steady-state trough concentrations within the target range. Target trough concentration range at steady state was defined 10–15 mg/L. The secondary outcome was predicted attainment of AUC_24h_ ≥400 mg^*^h/L. Their findings showed that 70.8% (102/144) of children with CDS-guided vancomycin attained the trough concentration target ranges, whereas only 37.5% (54/144) of children in the clinician-guided arm attained target ranges. Additionally, targeted AUC_24h_ was achieved in 93% (112/121) of occasions in the CDS-guided arm compared to 72% in the clinician-guided arm. Hughes et al. concluded that Bayesian software in a CDS tool improves the accuracy of PK attainment in individual pediatric patients. They argued that lower target attainment in the clinician-guided arm might be due to the hesitation of clinicians to recommend an adjusted dose above a certain amount, even when data would indicate that these changes are warranted.

### Supporting Studies With Simulations Studies to Optimize Dosing Regimens in Pediatric Patients

Several studies developed popPK models of aminoglycosides (amikacin, gentamicin and tobramycin) and vancomycin for a pediatric population, and performed model-based simulations to evaluate and compare dosing guidelines with respect to target concentration attainment (43–45). After applying a PK model in their population, Dao et al. suggested that vancomycin dosing strategy should be based on the combination of gestational age, postnatal/-menstrual age and serum creatinine concentration, since these covariates are associate with body composition, volume of distribution and renal function (43). A study by Mehrotra et al. reported that Monte Carlo vancomycin dosing simulations based on serum creatinine concentration have a greater likelihood of achieving trough target concentrations compared to four common dosing regimens n preterm and term neonates (44). External validation of model-based dosing for vancomycin, gentamicin and tobramycin resulted in a significant number (14.7–66.1%) of patients that attained sub-and supratherapeutic drug levels in critically ill children and neonates. This high interindividual variability might be associated with the incapability of the PK model to identify the source of variability (45). Based on these findings, the authors highlighted the necessity of external and real-world validation of guideline changes. Explaining the large intra- and inter-individual variability should be the main focus in future research to enhance drug exposure in critically ill children.

A popPK model of amikacin was developed by de Cock et al., and was used to evaluate current amikacin dosing regimens as suggested in textbooks at that time (2012) (46). This analysis illustrated that these dosing regimens commonly resulted in too high trough levels, associated with risk of toxicity. Consequently, a new model-based dosing regimen was evolved based on current body weight and PNA, and simulated in three typical patients. Findings showed that the model-based approach using birthweight and PNA was superior compared to guideline dosing regimens because it well-predicted amikacin clearance in neonates. Gonzalez et al. developed a population PK model in children to optimize clindamycin dosing in children (48). The relationship between PMA and clearance indicated that clindamycin dosing in neonates should be PMA based. Savic et al. used a modeling approach and simulations to evaluate rifampicin and levofloxacin dosing in order to attain target exposures (47). This study showed that higher rifampicin and levofloxacin dosages were required to reach target drug exposure.

### Model-Based Precision Dosing Implementation for Other Drugs in Pediatrics

Besides antibiotics, the improved outcomes as a result of MIPD implementation in pediatric populations had also been reported for other drugs, for example, sirolimus, fludarabine, doxapram, busulfan, morphine, carboplatin, or methotrexate (54–61). Mizuno et al. suggested that developed model-based dosing strategy could be utilized to explain the sirolimus exposure-response and clinical outcome relationships among pediatric population from neonates to adolescents (54). In a study of fludarabine, individualized MIPD most likely resulted in reduced morbidity-mortality and minimized toxicity in children (55). Additionally, model-based exposure which was integrated with the effect monitoring of drug therapy could improve doxapram treatment in pre-term infants (57). Furthermore, MIPD of busulfan combined with TDM utilizing a Bayesian prediction provides a considerable benefit compared to conventional guidelines for the attainment of target exposure in children receiving hematopoietic cell transplantation (HCT) (58). Morphine doses based on popPK model prevent over-dosing in infants with a PNA ≥10 days (60). Furthermore, model-informed Bayesian estimation was also compared to PK models alone and led to better morphine exposure in critically ill neonates and infants (61). In addition, population PK model of methotrexate was integrated into CDS tool which can be utilized to evaluate high exposure of methotrexate. Subsequently, this tool is able to inform the use of glucarpidase to reduce methotrexate plasma concentration (62).

In addition to pediatric populations, few studies also investigated MIPD in adults. Andersson et al. showed a significant benefit of busulfan TDM with MIPD over standard adult dosing in patients undergoing allogeneic HCT (56). Patients in the group with MIPD-guided dosing had a progression-free survival of 69.9%, compared to 11.2% in their fixed-dose counterparts (56). According to van Beek et al. TDM combined with MIPD of rifampicin is preferable to improve tuberculosis treatment compared to the linear regression strategy (37). Similarly, MIPD of warfarin in patients with heart valve enhanced the predictive performance of the maintenance dose of warfarin (63). Keutzer and Simonsson proposed that MIPD with PK information from minimally two drug concentrations can be applied to predict the optimal individual dose considering inter-occasion variability (64). In breast cancer patients treated with tamoxifen, MIPD was also considered as the more favorable strategy for attaining target concentrations than standard tamoxifen dosing (65).

## Discussion

While MIPD has the potential to improve the precision of antibiotic dosing in pediatric patients, the wide integration of MIPD for antibiotics in children into clinical practice is still scarce. The studies that we found are limited to vancomycin and amikacin. Based on the included studies, MIPD resulted in a better antibiotic exposure in children than the conventionally used dosing regimens. The improved target attainment might lead to enhanced efficacy and minimized toxicity. However, in none of the studies clinical outcomes and cost effectiveness were investigated.

MIPD is mainly used for drugs where adequate exposure at the start of therapy is critical and cannot be controlled by easy-to-measure clinical parameters (e.g., blood pressure or heart rate). Personalized dosing at the start of the treatment is crucial for effective antibiotic therapy. Therefore, MIPD in combination with TDM is desirable, so that optimal exposure is obtained both from the start and during treatment.

Three of the four eligible studies involved MIPD of vancomycin and one study amikacin. Several reasons may explain why research has been done mainly on vancomycin and less or not at all with other antibiotics. Firstly, vancomycin is well-studied because it is a first-line antibiotic to treat methicillin-resistant Staphylococcus aureus (MRSA) (66). Secondly, vancomycin has a narrow therapeutic index (67–69). Hence, guiding vancomycin dose with TDM is recommended in order to minimize the risk of nephrotoxicity and to guarantee successful therapeutic outcomes (70). Furthermore, vancomycin exposure is well-correlated with its response and toxicity, and these correlations are best predicted by the AUC_24h_/MIC ratio (71, 72). AUC_24h_ can be calculated using Bayesian estimations and cannot directly be translated from drug concentrations. Hughes et al. (42) and Leroux et al. (40) used trough concentration as the main outcome measurement, because it was the institutional target at the time of the study. Although current consensus guidelines recommend measuring trough vancomycin concentrations as a surrogate for the AUC_24h_, an AUC_24h_ estimation or Bayesian methods is superior, and therefore should be preferred in the MIPD approach.

For other commonly used antibiotic classes, such as aminoglycoside and beta-lactams, also can benefit from the utility of MIPD in children. Especially with aminoglycoside adequate dosing is necessary, given the toxic effects such as reversible nephrotoxicity and permanent ototoxicity (73, 74). A study by van Lent-Evers et al. suggested that model-based and TDM guided aminoglycosides dosing compared to non-guided TDM patients led to higher efficacy, shorter hospitalization and reduced nephrotoxicity (75). Accurate dosing of beta-lactams is also crucial for which MIPD could improve outcome, as these antibiotics are the cornerstone of anti-infective therapy in the critically ill patients. However, the majority of PK/PD and popPK model studies focus on agents where TDM is applied (30). Therefore, as expected, no MIPD studies of beta-lactams were performed as there is limited access to beta-lactam TDM services. Moreover, commonly used chromatographic methods are potential barriers to broad implementation in comparison with drugs easily quantifiable using immunoassay. Furthermore, PK and PD of these antibiotics in critically ill neonates and pediatric patients are poorly explored and sparse studies suggest that current dosing is frequently inadequate (30). There is a need to characterize population PK of commonly used beta-lactams in children, and patient characteristics associated with target attainment, in order to develop evidence-based dosing regimens. Additionally, the correlation between metabolism enzymes (genetic polymorphisms, drug-enzyme interaction) and other organ function parameters (e.g., CRP, IL6, biomarkers of renal clearance) should be explored as these parameters give the best description/reflection of the physical condition of critically ill children (15). This knowledge is essential for implementing MIPD to optimize exposure and improve clinical outcome in pediatric patients.

In the past decade, notable efforts have been put into the development of user-friendly, high-quality and highly-secured MIPD software tools (34). Another interesting development is the significant increase in the number of MIPD software tools with EHR integration capability to minimize data-entry burden (34). Frymoyer et al. (41) used a web-based dosing tool and Hughes et al. (42) integrated model-based dosing with a CDS tool and additional software to individualize dosing. Additionally, gentamicin model-based dosing in neonates and infants (neoGent) utilized a freely available MIPD tool which aids gentamicin TDM (76). The integration of a MIPD tool within the EHR can facilitate the adoption of precision dosing in routine clinical care (77). Kantasiripitak et al. evaluated 10 MIPD software tools and they concluded that improvements should still be made concerning EHR integration, standardization of software and model validation strategies, and prospective evidence for the software tools' clinical and cost benefits (34). AutoKinetics is one example of these tools and its functionality has been successfully expanded and adjusted for real time model informed precision antibiotic dosing at the bedside of critically ill patients (78).

The implementation of MIPD in routine practice can be challenging because it is involving patient's information, such as current characteristics, clinical data, and prior information on physiology to inform systems parameters. If data on one or several important parameters are missing for an individual patient, this will impair the translation by the model and deliver an adequate personalized dosing recommendation. In addition, routine genotypic testing and metabolic markers are rarely utilized to add information supporting individualized dosing (27). Yet, pharmacogenetics information can be incorporated with PK/PD model and TDM to bring MIPD at the bedside (79). To fully exploit the potential benefits of MIPD, the tools must be implemented in an easy-to-use framework for the team of healthcare providers. Importantly, the role of clinical pharmacists is considered as a success factor to implement MIPD (77). As suggested by Keizer et al., the struggles of MIPD from bench-to-bedside involves the many workflow steps as described in Figure 1 (28). In order to fully deploy MIPD in clinical practice, engaged clinicians as partners in implementing MIPD is essential for the development of intuitive tools for non-modelers (27). Furthermore, education and training for healthcare professionals are greatly needed to improve the comprehension about MIPD. Specifically, clinical pharmacists or pharmacologists have responsibilities to associate the link between PK/PD, pharmacometrics, system pharmacology, and clinical practice.

The MIPD approach is not an end in itself, but rather a tool or guide toward individualized medicine. It is associated with certain criteria that should be fulfilled, such as the existence of a well-defined concentration target and adequate allometric scaling methods, as the allometric approach explains only part of the variability in clearance (27). Furthermore, the sources of variability (e.g., age, organ failure, body weight, co-morbidity, or co-medication) in both the PK/PD target and MIC should be considered when using MIPD to assess target exposure. The use of a measured MIC obtained by a single MIC determination is debatable, since routine clinical laboratories cannot determine MICs with sufficient accuracy due to the inherent assay variation in the MIC test and the variation in any MIC determination (80). The epidemiological cut-off (ECOFF) of the presumed pathogens, can be used since the MIC is often unknown at the start of therapy. Although the ECOFF is in many situations similar to the clinical breakpoint, it is still important to closely evaluate the PK/PD target against the local drug resistance epidemiology.

Ultimately, the goal for MIPD is a bedside dashboard tool to determine adequate dosing at the start and during the treatment. This also includes real-time monitoring of disease progression and generating alerts for collecting PD data or covariates that are relevant. This can be of great additional value for treatment of vulnerable pediatric populations, where the clinical stakes are high for the treatment outcome and safety. Beside the need for widely developing and implementing MIPD tools at the point-of-care, it is also important to evaluate its clinical feasibility, efficacy and cost effectiveness. To do this, we still have to wait for results from randomized controlled trials investigating whether early MIPD in combination with TDM is superior to standard drug dosing strategies (81).

## Conclusion

This narrative review presents the current reported evidence for the clinical utility of MIPD of antibiotics in pediatric patients. The MIPD-approach poses a valid tool to predict future individual antibiotic exposure by means of Bayesian forecasting. We found only three studies of vancomycin and one study of amikacin concerning MIPD in children. Even though, those studies demonstrated that MIPD was superior compared to conventional dosing strategies with respect to the target attainment, the clinical utility of MIPD needs to be further confirmed for antibiotics, particularly aminoglycosides and beta-lactams.

## Author Contributions

AA and BK: conception and design. AA and EE: analysis, interpretation of the data, and wrote the first draft of the manuscript. All authors contributed to subsequent drafts and gave final approval of the version to be published.

## Conflict of Interest

The authors declare that the research was conducted in the absence of any commercial or financial relationships that could be construed as a potential conflict of interest.
