# Supplementary material for: Model-Informed Precision Dosing of Antibiotics in Pediatric Patients: A Narrative Review
Source: Front Pediatr. 2021 Feb 23;9:624639. doi: 10.3389/fped.2021.624639 (PMC7940353; doi:10.3389/fped.2021.624639)
Supplement: Supplementary file 1 [file Table_1.DOCX]

**Table S1.** Search strategy model-informed precision dosing of antibiotics in pediatric patients

| Medline All Ovid |
| --- |
| exp Anti-Bacterial Agents/ OR (antibiotic* OR anti-biotic* OR aminoglycosid* OR beta-lactam* OR polypeptid* OR penicillin* OR cephalosporin* OR fluoroquinolon* OR monobactam* OR carbapenem* OR macrolid* OR polymycin* OR tetracyclin*).ab,ti.) AND (Renal Elimination/ OR Half-Life/ OR Pharmacokinetics/ OR Drug Delivery Systems/ OR Hypoalbuminemia/ OR Protein Binding/ OR drug toxicity/ OR hypoalbuminemia/ OR protein binding/ OR plasma protein binding/ OR (((drug*) ADJ3 (accumulate* OR clearan* OR distribut* OR eliminate* OR excret* OR half-life OR time-curve OR activit* OR targeting OR intoxicat* OR toxicit*)) OR pharmacokinetic* OR pharmac*-kinetic* OR pharmacodynamic* OR pharmac*-dynamic* OR hypoalbumin* OR (protein* ADJ3 binding) OR (augment* ADJ3 (renal OR kidney) ADJ3 clearan*) OR (target* ADJ3 attainment*)).ab,ti.) AND (*Models, Statistical/ OR * Decision Support Systems, Clinical/ OR * Decision Support Techniques/ OR (model* OR (decision ADJ3 (support OR tool OR system*))).ti. OR ((develop* OR implement*) ADJ6 (model* OR decision-support* OR decision-tool* OR decision-system*)).ab,ti.) AND ("administration and dosage".fs. OR (dose OR dosage OR dosing).ti.) AND (exp child/ OR exp infant/ OR adolescent/ OR (child* OR infan* OR adolescen* OR pediatr* OR paediatr* OR neonat*).ab,ti.) NOT (exp animals/ NOT humans/) |
| Embase |
| 'antibiotic agent'/exp OR (antibiotic* OR anti-biotic* OR aminoglycosid* OR beta-lactam* OR polypeptid* OR penicillin* OR cephalosporin* OR fluoroquinolon* OR monobactam* OR carbapenem* OR macrolid* OR polymycin* OR tetracyclin*):ab,ti) AND ('drug accumulation'/de OR 'drug distribution'/de OR 'drug clearance'/exp OR 'renal clearance'/de OR 'drug elimination'/de OR 'drug excretion'/de OR 'drug half life'/exp OR 'plasma concentration-time curve'/de OR 'pharmacokinetics'/de OR 'pharmacodynamics'/de OR 'drug activity'/de OR 'drug targeting'/de OR 'drug toxicity and intoxication'/de OR 'drug intoxication'/de OR 'drug toxicity'/de OR 'hypoalbuminemia'/exp OR 'protein binding'/de OR 'plasma protein binding'/de OR (((drug*) NEAR/3 (accumulate* OR clearan* OR distribut* OR eliminate* OR excret* OR half-life OR time-curve OR activit* OR targeting OR intoxicat* OR toxicit*)) OR pharmacokinetic* OR pharmac*-kinetic* OR pharmacodynamic* OR pharmac*-dynamic* OR hypoalbumin* OR (protein* NEAR/3 binding) OR (augment* NEAR/3 (renal OR kidney) NEAR/3 clearan*) OR (target* NEAR/3 attainment*)):ab,ti) AND ('model'/exp/mj OR 'decision support system'/exp/mj OR (model* OR (decision NEAR/3 (support OR tool OR system*))):ti OR ((develop* OR implement*) NEAR/6 (model* OR decision-support* OR decision-tool* OR decision-system*)):ab,ti) AND ('dose'/mj OR 'drug dose'/exp OR (dose OR dosage OR dosing):ti) AND (juvenile/exp OR (child* OR infan* OR adolescen* OR pediatr* OR paediatr* OR neonat*):ab,ti) NOT ([animals]/lim NOT [humans]/lim) |
| Web of Science Core Collection |
| (((antibiotic* OR anti-biotic* OR aminoglycosid* OR beta-lactam* OR polypeptid* OR penicillin* OR cephalosporin* OR fluoroquinolon* OR monobactam* OR carbapenem* OR macrolid* OR polymycin* OR tetracyclin*)) AND ((((drug*) NEAR/2 (accumulate* OR clearan* OR distribut* OR eliminate* OR excret* OR half-life OR time-curve OR activit* OR targeting OR intoxicat* OR toxicit*)) OR pharmacokinetic* OR pharmac*-kinetic* OR pharmacodynamic* OR pharmac*-dynamic* OR hypoalbumin* OR (protein* NEAR/2 binding) OR (augment* NEAR/2 (renal OR kidney) NEAR/2 clearan*) OR (target* NEAR/2 attainment*))) AND ((dose OR dosage OR dosing)) AND ((child* OR infan* OR adolescen* OR pediatr* OR paediatr* OR neonat*))) AND (TI=(model* OR (decision NEAR/2 (support OR tool OR system*))) OR TS=((develop* OR implement*) NEAR/5 (model* OR decision-support* OR decision-tool* OR decision-system*))) |
